# Preliminary study of reliability of transcutaneous sensors in measuring intraabdominal pressure

**DOI:** 10.1038/s41598-022-12388-x

**Published:** 2022-05-18

**Authors:** Maša Kušar, Mihajlo Djokić, Srdjan Djordjević, Marija Hribernik, Simon Krašna, Blaž Trotovšek

**Affiliations:** 1 Department of Abdominal Surgery, University Medical Centre Ljubljana, Ljubljana, Slovenia; 2 Faculty of Medicine, University of Ljubljana, Ljubljana, Slovenia; 3 TMG-BMC Ltd., Ljubljana, Slovenia; 4 Faculty of Mechanical Engineering, University of Ljubljana, Ljubljana, Slovenia

**Keywords:** Sensors and probes, Gastrointestinal system, Musculoskeletal system, Biomedical engineering, Mechanical engineering, Signs and symptoms

## Abstract

Early recognition of elevated intraabdominal pressure (IAP) in critically ill patients is essential, since it can result in abdominal compartment syndrome, which is a life-threatening condition. The measurement of intravesical pressure is currently considered the gold standard for IAP assessment. Alternative methods have been proposed, where IAP assessment is based on measuring abdominal wall tension, which reflects the pressure in the abdominal cavity. The aim of this study was to evaluate the feasibility of using patch-like transcutaneous sensors to estimate changes in IAP, which could facilitate the monitoring of IAP in clinical practice. This study was performed with 30 patients during early postoperative care. All patients still had an indwelling urinary catheter postoperatively. Four wearable sensors were attached to the outer surface of the abdominal region to detect the changes in abdominal wall tension. Additionally, surface EMG was used to monitor the activity of the abdominal muscles. The thickness of the subcutaneous tissue was measured with ultrasound. Patients performed 4 cycles of the Valsalva manoeuvre, with a resting period in between (the minimal resting period was 30 s, with a prolongation as necessary to ensure that the fluid level in the measuring system had equilibrated). The IAP was estimated with intravesical pressure measurements during all resting periods and all Valsalva manoeuvres, while the sensors continuously measured changes in abdominal wall tension. The association between the subcutaneous thickness and tension changes on the surface and the intraabdominal pressure was statistically significant, but a large part of the variability was explained by individual patient factors. As a consequence, the predictions of IAP using transcutaneous sensors were not biased, but they were quite variable. The specificity of detecting intraabdominal pressure of 20 mmHg and above is 88%, with an NPV of 96%, while its sensitivity and PPV are currently far lower. There are inherent limitations of the chosen preliminary study design that directly caused the low sensitivity of our method as well as the poor agreement with the gold standard method; in spite of that, we have shown that these sensors have the potential to be used to monitor intraabdominal pressure. We are planning a study that would more closely resemble the intended clinical use and expect it to show more consistent results with a far smaller error.

## Introduction

Intraabdominal pressure (IAP) is the constant pressure present in the closed abdominal cavity^1^. IAP varies in the range of 5–7 mmHg in the healthy population^2^; in conditions associated with a slow increase in abdominal girth (obesity, pregnancy, ascites), these values can be chronically increased to 10–15 mmHg without a negative effect^1^. On the other hand, an acute increase in IAP causes a decrease in abdominal perfusion pressure (APP), defined as the difference between the mean arterial pressure (MAP) and IAP^3^, which leads to haemodynamic changes that can cause the dysfunction or failure of several organ systems.

Intraabdominal hypertension (IAH) is defined as a continuous rise in IAP above 12 mmHg^1^, and abdominal compartment syndrome (ACS) is defined as a continuous IAP above 20 mmHg with newly developed organ dysfunction^1,4^. Due to a possible clinical deterioration in patients with IAH and the need for preventing and treating IAH and ACS, IAP monitoring is required in all critically ill patients. Patients with high fluid replacement requirements, such as those with severe injuries and severe large burns, acute pancreatitis and other types of acute abdomen, are at especially high risk for the development of IAH and ACS^5–7^.

Currently, the most common (and gold standard) method of measuring IAP is a measurement of the hydrostatic pressure within the urinary bladder, which correlates fairly well with IAP when the appropriate technique is used^8^. However, there are several drawbacks to this method^9,10^. Of most importance, the precision of the method is insufficient; the 95% limits of agreement of values measured intravesically and laparoscopically are too wide for a useful measurement to be assumed ([−12.3 mmHg, 4.9 mmHg] at 50 mL of intravesical volume^8^) and outside of guidelines of acceptable accuracy^11^. Measurement reliability is also dependent on the staff conducting the procedure and this can lead to significant discrepancies between measurements^12^. Furthermore, the method is fairly labour-intensive and is usually only applied intermittently^13^, leading to a delayed diagnosis of increased IAP^7,14,15^. Other methods of measuring IAP have emerged, predominantly as gastric pressure measurements, which use similar principles to intravesical measurements^9^ and therefore presumably suffer from the same shortcomings.

Other approaches to measuring IAP have been proposed, such as measuring the abdominal perimeter^16^ or tension of the abdominal wall^17^. In the latter approach, a sensor is placed against the outer abdominal wall and measures the force and depth of the abdominal wall indentation. The abdominal wall acts as an elastic shell^18–22^, with the force of indentation proportional to the IAP^23^. Studies have been previously published that dealt with mathematical models and experimental approaches to measuring the connection between IAP and abdominal wall compliance^10,24–26^. Between them, the findings of the above studies are encouraging and imply that IAP could potentially be measured with a noninvasive transcutaneous method using this principle.

The sensor in our study^27^ detects changes in abdominal wall tension that can result from either muscle activity and/or changes in the pressure inside the abdominal cavity. The sensor was initially developed to measure muscle tension^27,28^, but its mechanism of action detects any tension changes in the elastic material under the surface in the longitudinal or transverse direction, which are not necessarily caused by muscle contraction. The changes in tension cause a change in the force of indentation and thereby the amount of bending of the sensor, which is registered by a piezoresistive silicon strain gauge element^28–30^. The electrical signal from the sensor is continuously logged.

We hypothesised that the use of the sensor could be changed in this way due to the difference in the shape of various muscle groups. The sensor was developed for use on the fusiform muscles of the extremities, such as the M. biceps brachii. Those muscles have clear muscle bellies that change shape and become rounder under tension, thereby increasing the tension on the strain gauge, allowing for an indirect estimation of the force produced. In contrast, the planar muscles of the trunk, such as the anterior abdominal musculature, display a far lesser degree of rounding. As a consequence, their activation alone should not trigger a significant change in the tension on the strain gauge, enabling the strain gauge to detect the change of shape of the entire abdominal wall.

The aim of this feasibility study was to determine whether tension changes in the abdominal wall, as measured by the transcutaneous sensor, could be used to assess changes in IAP.

## Methods

### Patients

We included 30 patients hospitalised in the Intermediate Care (IMC) unit of the Clinical Department for Abdominal Surgery, University Surgical Clinic, UMC Ljubljana. These were patients early in the postoperative course after abdominal surgery. We excluded patients who were physically unable to perform a Valsalva manoeuvre due to their general condition, as well as patients who were unwilling or unable to give informed consent. Furthermore, the study only included patients who still had a urinary catheter in place due to a medical indication to avoid the additional invasive procedure of placing it specifically for the study. Prior to the measurements, patients were instructed on the Valsalva manoeuvre, given the opportunity to practice it and provided with adequate analgesia to minimise any wound pain under tension. In this case, the Valsalva manoeuvre (i.e. forced exhalation against a closed airway that causes an increase in intraabdominal and intrathoracic pressure^31,32^) was performed without additional equipment. Patients were cued to perform it by mimicking a difficult bowel movement while holding their breath. The study itself was approved by the National Medical Ethics Committee of Slovenia and was conducted in accordance with the Declaration of Helsinki. Informed consent was obtained from participating patients.

### Equipment

We used transcutaneous sensors (MC-System, TMG-BMC, Ljubljana, Slovenia) to measure the tension on the abdominal wall. The sensor’s indenting tip protrudes towards the skin surface, where the force on the tip changes with the tension on the surface. The piezoresistive silicon force sensor with known sensitivity returns the output signal proportional to the indenting force. The association between tension on the surface and registered force at the tip of the sensor was previously established^27,29,30^.

Intravesical pressure was measured with an intraabdominal pressure measurement system (UnoMeter Abdo-Pressure, ConvaTec, UK). The sensor was placed between the patient’s urinary catheter and urine collection bag. In anuric and oliguric patients, the system can be filled with sterile saline solution to facilitate measurements in accordance with the manufacturer’s instructions. In cases where patients produce sufficient urine, a filling procedure is not needed. The zero value of the measurement scale is then aligned with the spina iliaca anterior superior, and the tubing is held vertically above that point to allow a direct reading of the pressure on the scale. The urinary catheters themselves were placed prior to including the patients in the study and were chosen according to their medical needs and physical characteristics. The IAP measurement system is designed to work with all available models of catheters and does not require a specific model or gauge.

The electric activity of the abdominal muscles was measured with a surface bipolar EMG electrode (Skintact F-301, Leonhard Lang GmbH, Austria).

The rectus muscle and subcutaneous layer thickness were measured by ultrasound with a linear probe in the 18–6 MHz frequency range (High Frequency Linear 8870, FlexFocus 500, BK Medical, Denmark).

### Measurements

**Figure 1:**
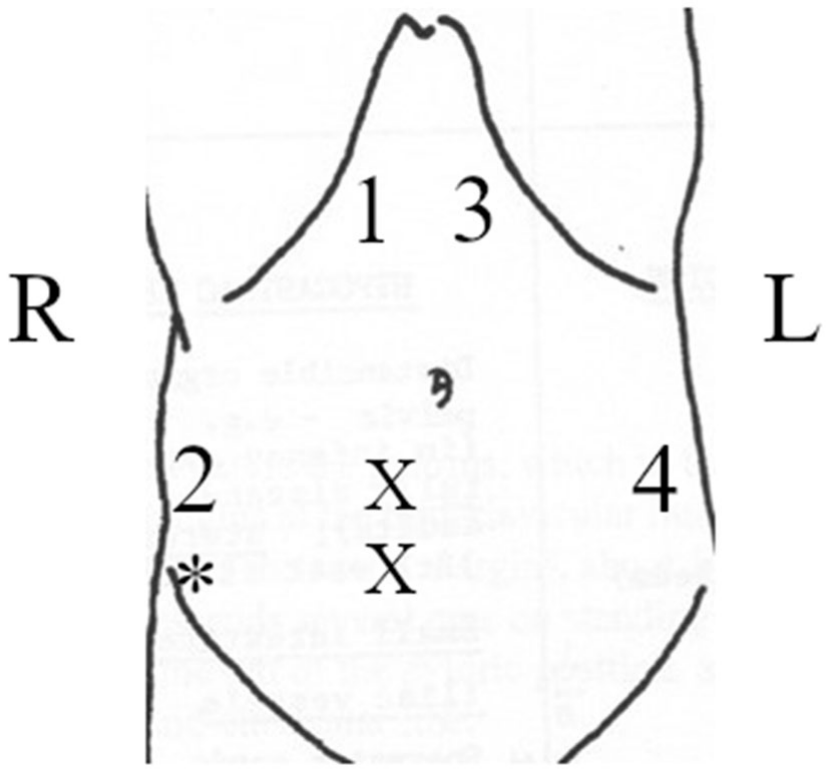
Idealised placement of MC and EMG sensors on the abdomen. Numbers 1–4 represent the placement of MC sensors; US measurements were conducted at 1. The double X shows the placement of both EMG electrodes. * shows the placement of the EMG ground electrode.

The volunteers were placed in a supine position with the bed levelled without inclination. Four sensors were placed (one on the cranial end of each rectus muscle and one on each oblique muscle, in the area between the anterior and middle axillary lines) and further secured with kinesiology tape. Two EMG sensors were placed on the caudal end of one of the rectus muscles, with the ground electrode placed near the iliac crest. The ideal positioning of the electrodes is shown in Fig. 1. The precise positions of the sensors (as well as the side of the EMG electrodes) were adjusted to accommodate the patients’ operative incisions, drains and ostomies (in patients with ostomies/drains/nonmedian incisions, the EMG electrodes were placed contralaterally, while the ipsilateral MC electrode positions were adjusted as needed, always overlying the corresponding muscle).

After attachment of the sensors, a 2 min period was used to neutralise the effect of skin viscoelasticity. The indentation depth of the transcutaneous sensor tip was set to 6 mm.

The IAP baseline, transcutaneous sensor signals and EMG signals were measured during calm breathing with relaxed abdominal muscles. The volunteers were then asked to perform the Valsalva manoeuvre 4 times, with at least 30 seconds between attempts to ensure a fall of IAP towards baseline. Patients were asked to maintain each attempt until the meniscus of urine/saline solution in the measurement system equilibrated, allowing for a reading of the intravesical IAP value on the scale. Peak intravesical IAP values under tension and resting intravesical IAP values were recorded for each attempt, resulting in 4 values under tension and 4 values during rest for each patient.

After removal of the measurement systems, ultrasound of the abdominal wall was performed. We recorded the maximum rectus thickness and overlying subcutaneous tissue thickness in the area of the upper rectus where sensor 1 had previously been placed. Whole abdominal wall thickness was calculated as the sum of the two.

### Signal processing

The EMG signal was recorded at a sampling rate of 2 kHz. After removing the offset, the raw EMG signal was bandpass filtered at 10–400 Hz with a 6th-order zero-lag Butterworth filter, full-wave rectified, and low-pass filtered with a 6th-order zero-lag Butterworth filter with a 1 Hz cut-off frequency to obtain the EMG linear envelope.

The MC sensor signals were recorded at a 1 kHz sampling rate. The raw MC signals were filtered with a low-pass 2 Hz 3rd-order zero-lag Butterworth filter.

**Figure 2:**
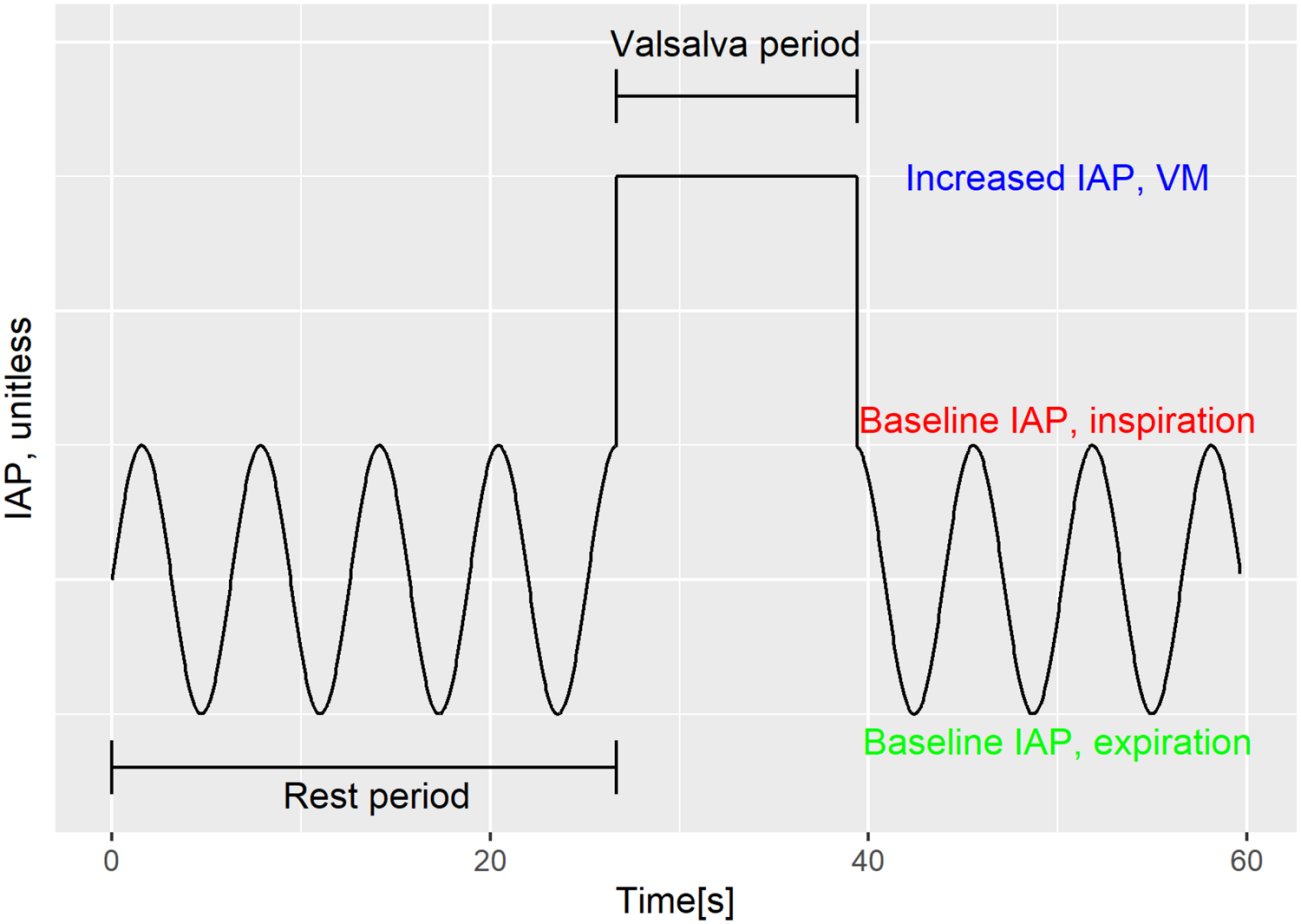
Ideal expected signal of a single MC sensor with an indication of how the rest and Valsalva periods are defined based on end-expiratory phases before and after the Valsalva manoeuvres.

As depicted in Fig. 2, the pauses between the four consecutive Valsalva manoeuvres were defined by the two end expiration phases nearest to the beginning and the end of the Valsalva manoeuvres, as identified by manual inspection. The measured intervals of the Valsalva manoeuvres did not include the period of the MC signal transition from the low to the increased level and vice versa. The average value of the MC signal during each period was recorded and used in subsequent analysis.

For each period, the average signal values from all 4 sensors were converted into units of mmHg using known calibration data and then averaged into a single value used in the model for IAP estimation.

### Statistical methods

For each patient, the intravesically-based and MC-based estimates of IAP were obtained 4 times at rest and 4 times during the Valsalva manoeuvre, for a total of 240 paired measurements. We used $\frac{3}{4}$ of the pooled data as a training set and the rest as a test set, ensuring reproducibility of the split by always using the same random seed within the R environment.

A linear mixed model that included transcutaneously estimated IAP values and subcutaneous tissue thickness as independent variables and intravesically measured IAP as the dependent variable was constructed. Models of various complexities were attempted (Table 1) and compared based on their Akaike information criterion (AIC) values and relative likelihoods (RL).

**Table 1:** Model components for three versions of a linear mixed model. The chosen model is outlined in bold.

	Fixed effects			Random effects	
	Average MC signal	Subcutis thickness	Signal*thickness interaction	Intercept	Slope
Full model	Yes	Yes	Yes	Yes	Yes
- Random slope	Yes	Yes	Yes	Yes	No
**- Fixed interaction**	**Yes**	**Yes**	**No**	**Yes**	**No**

AIC is a method that seeks to find the optimal model by penalising underfitting (poor goodness-of-fit) as well as overfitting (overcomplicated models). RL is one of the methods that estimates the plausibility of a given model compared to the most likely model. We chose the RL rather than the likelihood-ratio test because not all models were nested^33^. Once the final model was selected, population (using only fixed-effects coefficients) and individual (using both fixed-effects as well as random-effects coefficients) predictions were obtained and plotted together with the measured values to assess the degree of conformity.

After obtaining the model parameters, the model was used to make population-level predictions (i.e. using only fixed-effects coefficients) of IAP from the MC pressures and subcutis thickness in the test set.

From this, a Bland-Altman graph^34–36^ was drawn to assess the suitability of our measurements to estimate the IAP values. Furthermore, the measurements in the test set were used to test the predictive ability of the transcutaneous measurements in determining whether IAP was>20 mmHg. To do this, an ROC curve was plotted, the AUC was calculated and the specificity, sensitivity, positive predictive value (PPV) and negative predictive value (NPV) of the MC measurement to predict IAP>20 mmHg were calculated.

Bland-Altman analysis can be modified to take into account repeated measurements. In our analysis we chose to use a simple Bland-Altman analysis, as if the measurements were all independent of each other. We made this decision based on two facts. On one hand, we have not found another example in the literature on the subject of a modified Bland-Altman (or any other) analysis. Fusco et al.^8^ had hierarchical data grouped on three levels (by subject, by bladder volume and by insufflated pressure) and used simple analysis for both regression and Bland-Altman analyses. While this is methodologically questionable, we chose the same approach to maintain the ability to compare the results. Furthermore, in our case, the dataset included in the Bland-Altman analysis was arguably no longer hierarchical. The estimated IAP values from the MC measurements were population level estimates, which no longer include the random terms, and are therefore not clustered within subjects (ie. all estimates done with the same slope and intercept coefficients). While a single subject’s generated pressures may have been clustered around a single point or within a limited range of pressure, the MC estimated pressures no longer included subject-level random terms.

All statistical analyses and data visualisation were conducted with R (v 3.6.3)^37^ in RStudio (v 1.1.419)^38^, using base R and the packages lme4 (v 1.1-23)^39^, lattice (v 0.20.41)^40^, nlme (v 3.1–148)^41^, car (v 3.0–8)^42^, MuMIn (v 1.43.17)^43^, knitr (v 1.29)^44^, kableExtra (v 1.1.0)^45^, ROCR (v 1.0-11)^46^, ggplot2 (v 3.3.2)^47^ and their dependent packages.

## Results

The patients’ characteristics are presented in Table 2. The average values of MC signals from both recti, calculated for different periods of the protocol, were correlated with each other (r = 0.77), but but not closely enough to justify averaging them into a single measurement. Furthermore, as shown in Table 3, the pattern of correlations between the muscles was inconsistent and not necessarily in line with what is biologically expected simply as a result of muscle activation. We attribute this to the voluntary nature of the contractions which is affected by the actual pain the patients experience, as well as the fear of pain they might experience. Seeing that both rectus muscles are well-correlated is expected, as they are similarly affected by midline incisions and minimally, or not at all, affected by off-centre interventions. Furthermore, they are usually activated together, whether voluntarily or as a reflex guarding against pain. Conversely, lateral incisions and drains cause localised one-sided pain that can inhibit activation of the ipsilateral oblique musculature. Even ostomies, though traditionally placed within one of the rectus muscles, would have a greater effect on the correlation between the obliqui than the recti. This is because the pain of a fresh ostomy would inhibit the activation of both recti (which we cannot activate separately) and would have a greater effect on the ipsilateral oblique muscles (which can be activated separately from the contralateral oblique muscles).

**Table 2:** Baselines patient characteristics.

Characteristic	Mean	Range
Age [years]	66	46–81
Height [cm]	170	152–187
Weight [kg]	77	52–105
BMI	26.4	18.4–41.0
Subcutaneous fat thickness [mm]	12.0	1.6–24.6
Rectus muscle thickness [mm]	9.6	4.3–14.0
Abdominal wall thickness [mm]	21.7	9.7–34.8

**Table 3:** Estimated correlation coefficients between the signal outputs obtained from various pairs of MC sensors.

Pair of muscles	Correlation
Right muscles	0.73
Left muscles	−0.20
Mm. obliqui	−0.80
Mm. recti	0.77

The subcutis thickness was highly correlated with the whole abdominal wall thickness (0.91) and moderately correlated with BMI (0.59). It was also moderately negatively correlated with the average of all 4 MC signals (−0.55), which was expected, as the subcutaneous layer dampens the signal of the changes in the ”true” muscular abdominal wall. As such, it seemed reasonable to include it in the model as a measure of the composition of the abdominal wall, which was expected to influence the measured values.

**Figure 3:**
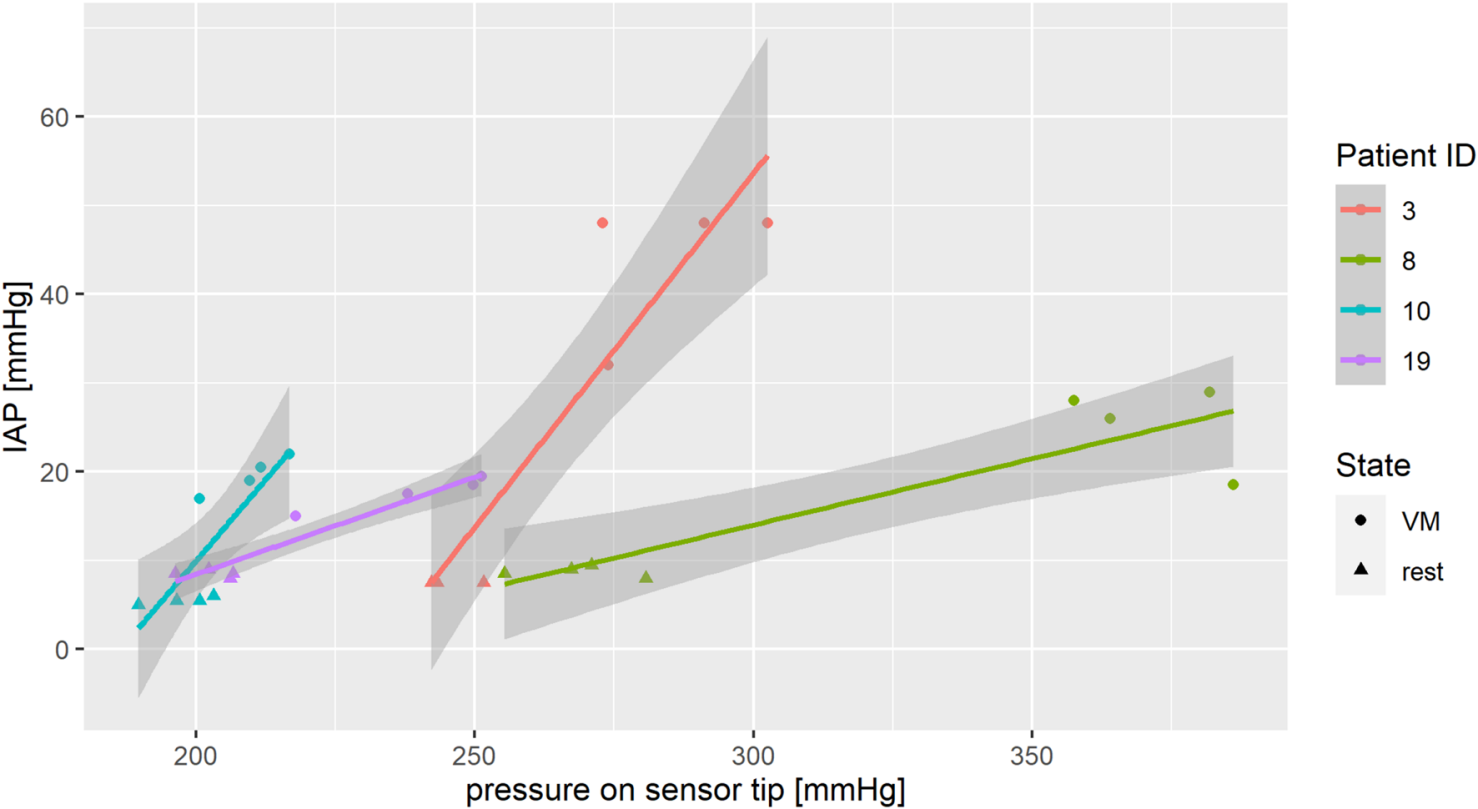
Intravesical and calibrated MC measurements of pressure in selected patients. Note: the MC values show the raw values of pressure on the sensor tip and have not yet been modelled to estimate the IAP.

Visually, the data appear to be consistent within the subjects, but they were highly variable between them, further supporting the use of a mixed model. As seen in Fig. 3, there seems to be an approximately linear relation between intravesical pressure and pressure on the sensor tip within each subject, but the slopes and intercepts of the regression lines vary. There are clear outliers in the data, but the positive correlation is nevertheless visible.

### Model performance

Three versions of the linear mixed model were tested; each included the subcutis thickness and average MC signal values as predictor variables and a random intercept term (to account for the non-independent repeated measurements within each subject). The models differed among each other in the inclusion of an interaction term between both fixed effects and a random slope term (additive to the fixed slope term for the MC signal values). Details of the models as well as their performance are summarised in Tables 1 and 4. The performance is shown in terms of the Akaike information criterion (AIC) and the relative likelihood of each model (RL). The latter is interpreted as the relative likelihood that the given model is the one that minimizes the information loss when compared to the model with the smallest AIC.

**Table 4:** Model performance for three versions of a linear mixed model.

	AIC	RL
Full model	193	0.001
- Random slope	189	0.008
- Random intercept	179	1

It is clear from Table 4 that the more complex two models are very unlikely to minimize information loss, so we assumed the simplest model to be the best representation of the data at hand.

The marginal $R^2$ value of the chosen model is 0.409, while the conditional $R^2$ is 0.854, indicating that 85.4% of the observed variability is explained by the model, with 40.9% explained by the fixed factors (transcutaneously estimated IAP and thickness of the subcutaneous tissue) alone.

The regression coefficient for the transcutaneously estimated IAP values is 0.0076 (95% CI [0.0056, 0.0096]), with $p<0.0001$; the regression coefficient for subcutaneous tissue thickness is 0.069 (95% CI [0.032, 0.107]), with p=0.0008. Both the transcutaneously estimated IAP values and the subcutaneous tissue thickness are therefore significantly associated with the intravesically measured IAP and and therefore were used in the final model to estimate the IAP values in the test set (the population-level predictions from the chosen mixed model).

### Model utility

**Figure 4:**
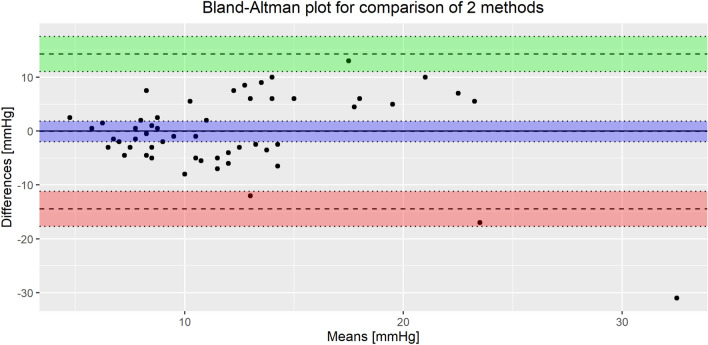
Bland-Altman plot of the agreement between the intravesical and transcutaneous methods of IAP measurement with corresponding 95% confidence intervals of estimates of bias and limits of agreement. The difference between methods varies more widely in very high pressures, providing an indication that the imprecision may be at least partially a result of the mechanism by which the high pressure is generated.

Using the selected model, we made population-level predictions (that is, using only fixed factors) on the test set and used these to construct a Bland-Altman plot to compare the IAP values obtained from both measurements. The results of the comparison can be found in Table 5 and Fig. 4. The estimates from our sensors are unbiased, but the limits of agreement are currently too wide to be clinically useful for continuous measurement.

**Table 5:** Comparison between transcutaneous and intravesical methods: Bland-Altman analysis and mean absolute error.

Characteristic	Estimate	95% CI
Bias	− 0.0667	−1.96 to 1.83
Upper limit of agreement	14.31	11.06 to 17.57
Lower limit of agreement	− 14.45	− 17.70 to − 11.19
Mean absolute error	5.53	− 3.80 to 14.87

We extended the analysis to include a binary classification of a very high IAP; the chosen cut-off point was IAP=20 mmHg. This cut-off point is clinically significant, as patients who do not reach this IAP are usually not candidates for surgical decompression. The results of this analysis are included in Table 6. The accuracy, specificity and negative predictive value of such a classification are high and show clinical potential in identifying patients who do not require invasive surgical decompression.

**Table 6:** Measures of accuracy of binary classifications of MC-based IAP estimates.

Characteristic	Estimate
Specificity	0.88
Sensitivity	0.33
Positive predictive value	0.125
Negative predictive value	0.96
Accuracy	0.85
Area under the ROC curve	0.81

It should be pointed out that the positive and negative predictive values are dependant on the prevalence of the observed phenomenon in the studied population. Given the low prevalence of IAP ≥ 20 mmHg in our sample, a high negative predictive value is not unusual and would decrease in a sample with a larger prevalence of high IAP. However, the sample prevalence of high IAP is in agreement with the prevalence of such extreme values in the ICU population. While increases of IAP in critically ill patients are common, extreme increases over 20mmHg with the concomitant possible need for surgical treatment, are rare. Thus, the validity of the prediction cannot be refuted on the basis of this consideration.

## Discussion

Previous studies have shown that tension on the abdominal wall is correlated with IAP^23^, but ours is the first to our knowledge to attempt an internal validation of the accuracy of a non-invasive method of IAP estimation based on abdominal wall tension sensors. Our trial showed that transcutaneously estimated IAP is significantly associated with intravesically measured IAP, and our sensors give an unbiased estimate of IAP compared to the intravesical measurements, but the limits of agreement between the methods are wide.

However, we believe that the wide limits of agreement can be explained by two factors: the insufficient precision of the standard method as well as the study design, where the increases in IAP were voluntarily intrinsically generated.

### Comparison to the gold standard method

When a standard method has poor repeatability, any new method compared to it is expected to demonstrate wide limits of agreement even in the case of perfect agreement with the true value, which is a direct result of the poor repeatability of the standard method^35^. The repeatability of the reference method unfortunately cannot be estimated from the available data in Fusco et al.^8^, so the effect of this cannot be directly estimated. However, with the intravesical method having very wide 95% limits of agreement with laparoscopic values ([−12.3 mmHg, 4.9 mmHg] at 50 mL bladder volume^8^), there is a clear issue with its precision. Given this shortcoming of the intravesical method, the limits of agreement of the transcutaneous measurements compared to intravesical measurements were expected to be wide.

Furthermore, a previously performed study has shown fairly wide 95% limits of agreement in assessing interobserver repeatability^12^ of intravesical measurements of IAP. Note that the study design in Kimball et al.^12^ only observed variability between observers in reading the results of the measurement while completely excluding the variability in setting up the measurement, which is the part of the procedure most likely to lead to inconsistent measurements. Even with that underestimation of variability, the reproducibility of the intravesical measurements is too low for reliable clinical use.

Between the wide limits of agreement and the poor interobserver repeatability of the gold standard method, we believe that a development of a possible new method is warranted in spite of the existence of an already established method.

### Factors related to the study design

Furthermore, we think a part of our wide limits of agreement is a direct result of the experimental setup and would be greatly diminished in a more realistic clinical scenario.

To compare the transcutaneous and intravesical values at different IAPs, the patients had to generate increases in IAP by means of the Valsalva manoeuvre. We intentionally chose this experimental setup to conduct the initial test without including more severely ill patients. We had two concerns with this. One was that the voluntary muscle contraction itself, without affecting the IAP, would affect the transcutaneous signals, potentially to a greater degree than the actual IAP change itself. This would mean we would be unable to use our measurements to reliably assess changes in IAP alone. This would be in keeping with the original use of the sensors; they were developed to measure the force in the underlying muscle rather than intratissue pressures^29,30^. The other concern was that a voluntary IAP increase does not cause a consistent change in the shape and tension of the abdominal wall, as the activated muscles counter the effect of increased IAP on the abdominal wall shape. As an example, the activity of the diaphragm and the intercostal muscles can influence IAP on a voluntary basis^15,48^, but this might be masked by the concurrent activation of the abdominal muscles that would prevent a change in the shape of the abdominal wall. This could reduce the effect of IAP changes on the tension in the abdominal wall that is detected by the sensor.

**Figure 5:**
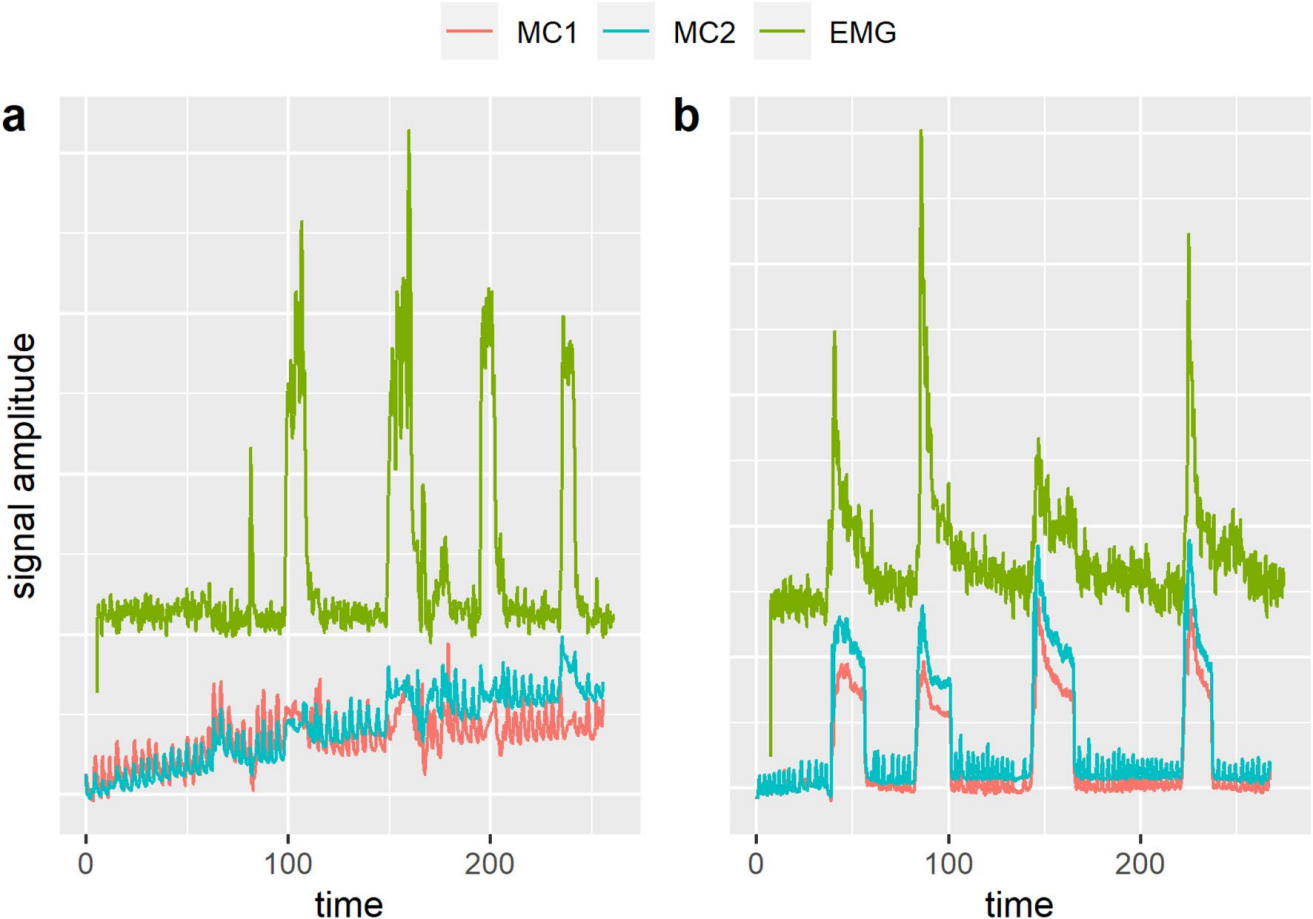
Exemplary recordings of EMG and MC signals from two patients. (**a**) Patient without rise in IAP - unsuccessful VM. (**b**) Patient with rise in IAP - successful VM. On the left, it is clear from the EMG that the patient was activating the musculature, but the MC signals only show a disturbance in the respiratory oscillations without a significant and/or sustained change in the mean MC signal. Note: The continuously rising MC signal seen in figure 5a was also observed in some of the patients who have managed to raise their IAP, where it was most pronounced during the rest phases. We believe this to be a physical artifact of the sensor, possibly related to a too short waiting period before the measurement, as the residual skin viscoelasticity after attachment of the sensors could be causing the drift.

The first concern was sufficiently addressed during the course of the trial. There were several patients who activated their muscle wall, as recorded with EMG, which showed the same patterns as all other patients, but they were unable to produce an increase in measured intravesical pressure with this activity ; the EMG and MC signal traces of one such patient can be seen in Fig. 5). While this was occurring, their transcutaneous pressure reading showed a cessation of normal breathing-related signal oscillation, but the overall mean signal did not change. The remaining oscillations may be an indication of an incorrectly performed Valsalva manoeuvre, which could explain the lack of IAP increase with clear muscle activation. This showed that the sensor did not respond to muscle activation that occurred without changes in IAP. This leads us to believe that the detected changes in the signal in the other patients were truly a result of raised IAP and not interference caused by muscle activity.

We believe this difference in IAP behaviour may be a reflection of the difference between the Valsalva manoeuvre (an expiratory effort) and abdominal straining (inspiratory effort)^49,50^, as some of the subjects may have inadvertently performed the latter.

Meanwhile, the variability of abdominal wall shape changes has certainly been shown to be an issue. Despite this severe limitation, the results show that changes in intravesically measured IAP are significantly associated with changes in transcutaneously measured IAP, as well as the thickness of the subcutaneous layer of the individual. However, the effect of differences among individual patients was rather large in this experimental setting (as shown by the low marginal $R^2$). This led to wide limits of agreement in transcutaneously estimated IAP that would make these measurements clinically irrelevant if this were the expected clinical performance of the sensor. However, the foreseen clinical use of these sensors is in severely ill or injured patients at risk of developing ACS. In those patients, the increase in IAP is generated within the abdominal cavity and not from voluntary activity of the abdominal wall. With this large source of variability eliminated, it is our belief that the marginal $R^2$ would increase significantly. This would lead to population estimates that were in much greater agreement with the individual estimates and intravesically measured values. We have already shown that transcutaneous measurements are unbiased when compared to intravesical pressure measurements. With the reduced variability of a clinically more realistic setting and a direct comparison to true laparoscopically measured values, it is expected that the limits of agreement of the predictions would narrow considerably.

### Other concerns

A further limitation to the results of the study is the significant effect of subcutaneous tissue thickness on the amplitude of the MC signal and its change. This is a factor that cannot be controlled in the clinical situation but that may limit the usefulness of the MC sensor in patients with a higher subcutaneous tissue thickness. One way to account for this may be the variable size of the spherical tip and indentation depth of the sensor. This consideration could also be addressed in future studies.

The large variability between measurements in different locations, especially between the obliqui, is another possible source of measurement error. All 4 measurement locations were initially included in the study design, since we had no reason to believe that either the obliqui or recti measurements would be superior in measuring IAP. Table 3 shows that the consistency of obliqui measurements is inferior in our study. Including all four measurements separately was not possible, as the number of model variables would have been too large given the number of subjects. Another possibility would have been to exclude the obliqui measurements post-hoc to reduce the variability in measurements. We have chosen to keep them in the overall signal average to avoid modifying the study design in search of lower p values. Furthermore, this inconsistency may very well be an artifact of the incisions that will be annulled in further studies on sedated patients. If this is the case, removing the obliqui measurements in this study would have resulted in an over-fitted model that would be even harder to replicate successfully. If the obliqui measurements’ inconsistency persists even in better controlled circumstances, we will certainly consider this sufficient reason to doubt the value of these measurements and exclude them from further sensor development.

As seen in Fig. 5a, there was an upward drift of baseline MC signal throughout the experiment in some patients. Based on the concurrent intravesical measurements, we believe this can mainly be attributed to two factors. First, a portion of these subjects also had a slight increase in intravesically measured pressures. While this was occurring, the rest periods were prolonged as needed until the measured pressure stabilised, before reading off the value and starting the next VM. In these subjects, we believe the true resting IAP may have increased slightly (no more than 2 mmHg), perhaps as a result of anticipation of the next VM. On the other hand, we observed an approximately linear upward drift in some patients whose intravesically measured resting pressures were identical. This is very likely a sensor artifact. We believe the waiting period between sensor attachment and the measurements may have been too short, leading to residual drift due to the skin viscoelasticity. The first of these concerns will clearly be addressed by the altered study design described below. The second concern will be addressed by prolonging the waiting times and would be expected to be less of an issue in prolonged continuous use.

We plan to re-evaluate all of these limitations in a setting where the IAP increase is not voluntary. An involuntary change in IAP (ventilator-induced in ICU patients or insufflator-induced during laparoscopy, both in patients treated with neuromuscular relaxants) would allow the shape of the abdominal wall to change freely with the IAP change. This situation would better correspond to the realistic clinical situation in which IAP needs to be measured. In such a scenario, we expect an increase in the observed changes in transcutaneous signals at the same levels of the IAP change, thereby increasing the relative size of the fixed effects in the model. At the same time, the MC signal changes should prove to be more uniform across patients, thereby decreasing the relative size of the random effects in the model. Due to this, we expect both the marginal and the conditional $R^2$ value to increase, as our model (and in particular its fixed effects) explains a larger part of the variability. Furthermore, we will no longer be limited to assessing the performance of the transcutaneous method compared to the intravesical. Instead, we will be able to directly compare both methods’ accuracy compared to the true laparoscopic values. As a result, the Bland-Altman plot is likely to change to a more favourable configuration with narrower limits of agreement. With the decrease in transcutaneous estimation variability caused by random effects, we also expect the sensitivity, PPV and AUC to increase, possibly achieving a clinically more acceptable prediction performance.

Given the wide limits of agreement (to the point of having questionable clinical value) of the intravesical method with laparoscopic measurement, continued attempts to find and fully assess an alternative method seem justifiable.
